# Tensors all around us

**DOI:** 10.3325/cmj.2019.60.369

**Published:** 2019-08

**Authors:** Branimir K. Hackenberger

**Affiliations:** Department of Biology, Josip Juraj Strossmayer University, Osijek, Croatia *hackenberger@biologija.unios.hr*

Advanced artificial intelligence (AI) technologies have become so integrated in our daily lives that they no longer fascinate us. Online database search engines, image search engines with embedded recognition systems, device management by voice, have all become normal and common. However, every time I use Shazam, I am amazed at its speed in identifying music, its version and author. Sometimes in less than a second!

It seems impossible to keep up with all the trends when it comes to the technology of data processing. But maybe we should not bother. The computers will follow the trends for us, using AI algorithms. I do not know how many people have noticed it but making just a few Google searches on a certain topic leads to notifications on that topic appearing on other pages. For example, if you google “non-steroidal analgesics,” do not be surprised to see messages and ads about these products on social networks or blogs.

In the same way, our computers will track very closely the kind of data we have, the kind of results we need, and the methods we use. The time is coming rapidly when, based on trend screening, computers will optimize the methodology and suggest the best for us. A positive aspect to AI is the openness and availability of its core technologies. Machine and deep learning, two basic technologies necessary for the development of AI systems, are no longer a curiosity but have become standards in the processing of large data amounts. Predictive, as well as analytical, systems based on neural networks are a common thing. Such a dizzying development of AI technology has been made possible by the code openness and simplifying the complicated programming methods.

The programming language Python and statistical environment R (RStudio) are today indispensable tools in data processing. While these two free software tools are a subject of controversy, it cannot be denied that they have experienced a surge in popularity and are significantly represented in all areas that use data processing. However, this phenomenon may be a topic of another column. The openness of Python and R enabled the rapid implementation of AI technology, as well as its utilization in a range of applications. A crucial step that allowed this to happen occurred in 2015, when Google Brain, Google's AI research team, released a free and open-source software library named TensorFlow. In 2017, its first stable version became available. The most important parts of the library, computational ones, are written in C ++, while Python is used as an excellent higher-level language management platform.

The emergence of TensorFlow has facilitated and accelerated the development of applications whose functioning requires machine, ie, deep learning. Just as the development of machine and deep learning theory has accelerated the development of AI theory, so has the emergence of the TensorFlow library accelerated the development of AI applications. However, TensorFlow is not the only framework for implementing machine and deep learning. The first such framework was Torch, released in 2002, followed by Theano in 2007, Caffe in 2013, and Keras in 2015. Of course, other free frameworks, such as BigDl, Chainer, OpenNN, or commercial ones, such as Intel Math Kernel Library, Deep Learning Toolbox for MATLAB, and Neural Designer have also evolved.

**Figure 1:**
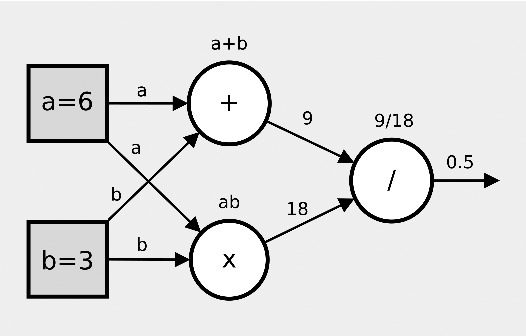
A graph containing two input variables and three operations giving one output.

The name “tensor” refers to an edited set of data, usually numbers. We call the zero-order tensor a scalar. Each individual number is a zero-order tensor. First-order tensors are vectors. We can say that every finite set of numbers is a first-order tensor. Third-order tensors are arrays, etc. TensorFlow works with data organized into tensors, but the dimension of the tensor is not important. There is no limit to data complexity. TensorFlow does not perform operations with tensors immediately but stores them in special data structures, called graphs. This allows the so-called datastream programming, and each computation can be displayed as a graph, which facilitates model analysis and correction. Figure 1 shows a graph containing two input variables and three operations giving one output. In other words, this graph shows the flow of calculating output = (6 + 3) / (6 * 3). In this simplified example, graph input data are constants, however, input data may be variables, and machine/deep learning requires a specific form of frames of input data – placeholders. Placeholders are actually empty nodes of a graph that can be used to enter data from the outside, such as from a database, as needed. The variables entered and the data flowing between the nodes do not have to be simple scalars as in this example but can be tensors of any dimension. The first advantage of this computation concept is apparent from Figure 1. Namely, it is more than obvious that two operations (multiplication and addition) can be performed simultaneously, ie, in parallel. This is why most machine/deep learning frameworks extensively use parallel computing hardware. This is also the reason why, in addition to Python and C ++, many frameworks use CUDA. CUDA is a parallel computing platform, created by Nvidia, that allows the use of CUDA-enabled graphics processing units (GPUs). Another important advantage of this concept is portability. Namely, a model written in Python can be easily converted to any other language, such as C ++.

**Figure 2:**
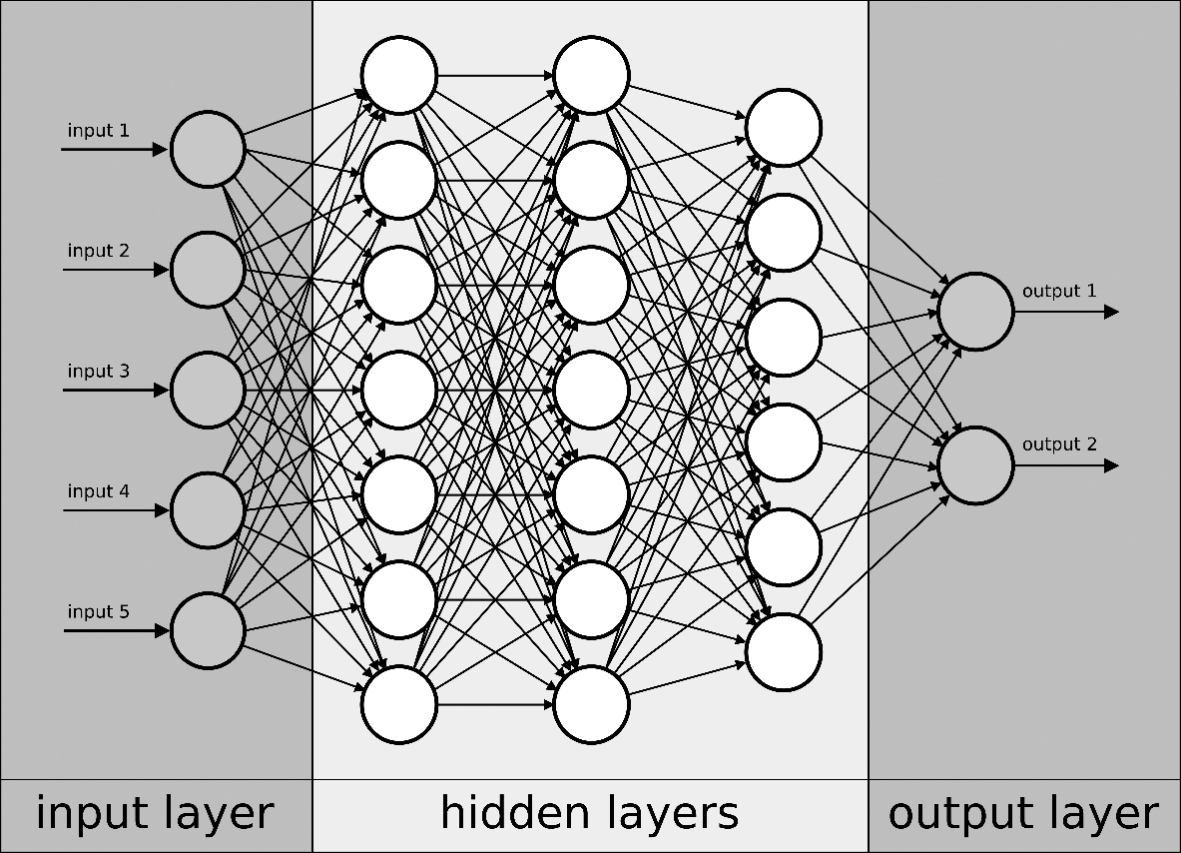
A simplified flowchart of an artificial neural network.

The entire work with machine/deep learning systems is based on two steps. The first step is the construction of a model, ie, a computational graph, and the second step is the so-called session. A session is nothing else than a runtime in which the data flow through nodes, ie, operations, is initiated. A simplified flowchart of an artificial neural network (ANN) is shown in Figure 2.

In 2017, Google decided to support Keras in TensorFlow's core library. It was a significant decision that extended Keras’ life as a kind of interface to TensorFlow. At the same time, the combination of TensorFlow and Keras resulted in a whole machine/deep learning framework, which makes programming much easier. Arguably, Keras has become a high level management environment (application programming interface, API) for TensorFlow.

If you want to use the statistical environment R to work with TensorFlow and Keras, there are at least five different models available at the moment: 1. Convoluted Neural Networks, 2. Multi-Layer Perceptrons, 3. Recurrent Neural Networks, 4. Skip-Gram Models, and 5. pre-trained models. Working directly in Python has almost no restrictions.

Currently, TensorFlow (including Keras) is the biggest competitor to the following three machine learning frameworks: PyTorch, MXNet, and Microsoft Cognitive Toolkit. PyTorch and TensorFlow, in addition to being written in Python, share many similarities. First of all, there is a lot of interactivity in application development and a predefined hardware acceleration component. PyTorch is the ideal frame for smaller projects and simpler workflows, while TensorFlow is far better for larger and more complex projects. MXNet is an extremely flexible machine/deep learning framework that allows the addition of new hardware components to an already functional ready-made system (in IT jargon, this framework can scale to multiple CPUs and GPUs and multiple machines). Although MXNet supports many languages (Python, C ++, R, JavaScript, Julia, etc.), working with its APIs is more complicated than working with TensorFlow's APIs. Microsoft Cognitive Toolkit, previously known as CNTK, is more focused on deep learning than TensorFlow. Although in some aspects it is much faster and although there are APIs for a whole range of languages (Python, Java, C ++, C #), the learning curve in this framework is far less steep than in TensorFlow. An overview of the commonly used and actively developed and maintained frameworks is given in Table 1.

**Table 1:** Commonly used, actively developed, and maintained machine/deep learning frames

Software	Released	License	Language	Interface	Graphics processing units support
**PlaidML**	2017	AGPL	Python, C++, OpenCL	Python, C++	No
**BigDL**	2016	Apache	Scala	Scala, Python	No
**Microsoft Cognitive Toolkit**	2016	Apache	C++	Python (Keras), C++, BrainScript	Yes
**PyTorch**	2016	BSD	Python, C, C++, CUDA	Python, C++	Yes
**Apache MXNet**	2015	Apache	Small C++ core library	Python, R, C++, Julia, Matlab, JavaScript, Go, Scala, Perl	Yes
**Intel Data Analytics Acceleration Library**	2015	Apache	C++, Python, Java	Python, C++, Java	No
**Keras**	2015	MIT	Python	Python, R	Yes
**TensorFlow**	2015	Apache	C++, Python, CUDA	Python (Keras), R, C/C++, Java, Go, JavaScript, Julia, Swift	Yes
**Caffe**	2013	BSD	C++	Python, MATLAB, C++	Yes
**Theano**	2007	BSD	Python	Python (Keras)	Yes
**Torch**	2002	BSD	C, Lua	Lua, LuaJIT, C, C++/OpenCL	Yes

Anyone who chooses to use one of these machine/deep learning frameworks or test their models has open databases or data sets available. Table 2 shows some of the most interesting free data sets with medical data. In addition to these databases, there is a range of free data sets useful for calibrating or testing models for processing images, sounds, biometrics, etc. In programming languages, the Modified National Institute of Standards and Technology database data sets are often used as an example. In addition to the data sets, there is a number of tutorials and online courses that allow beginners to very quickly find and create their own models.

**Table 2:** Free medical data sets available on the internet suitable for making, training, and testing machine/deep learning models

Name of data set	Released	Description	Instances
MHSMA (Modified Human Sperm Morphology Analysis Data set)	2019	Human sperm images from 235 patients with male factor infertility, labeled for normal or abnormal sperm acrosome, head, vacuole, and tail.	1540
Parkinson’s Data set with replicated acoustic features Data Set	2019	Acoustic features extracted from 3 voice recording replications of the sustained /a/ phonation for each one of the 80 subjects (40 of them with Parkinson’s disease).	240
Breast Cancer Coimbra Data Set	2018	Clinical features were observed or measured for 64 patients with breast cancer and 52 healthy controls.	116
DeepLesion	2018	Over 32 000 annotated lesions identified on CT images.	>32,000
Drug Review Data set (Druglib.com) Data Set	2018	Data set with patient reviews on specific drugs along with related conditions. Reviews and ratings are grouped into reports on the three aspects: benefits, side effects, and overall comment.	4143
Drug Review Data set (Drugs.com) Data Set	2018	Database with patient reviews on specific drugs along with related conditions and a 10 star patient rating reflecting overall patient satisfaction.	21,5063
Ultrasonic flowmeter diagnostics Data Set	2018	Fault diagnosis of four liquid ultrasonic flowmeters	540
WESAD (Wearable Stress and Affect Detection) Data Set	2018	Data of 15 subjects during a stress-affect laboratory study, while wearing physiological and motion sensors.	6,3E+07
Parkinson’s Vision-Based Pose Estimation Data set	2017	2D human pose estimates of Parkinson’s patients performing a variety of tasks.	134
Mesothelioma Data set	2016	Mesothelioma patient data.	324
Tox21 Data set	2016	Prediction of outcome of biological assays.	12,707
Diabetes 130-US hospitals for years 1999–2008 Data set	2014	9 y of readmission data across 130 US hospitals for patients with diabetes.	100,000
Diabetic Retinopathy Debrecen Data set	2014	Features extracted from images of eyes with and without diabetic retinopathy.	1151
National Survey on Drug Use and Health	2012	Large scale survey on health and drug use in the United States.	55,268
KEGG Metabolic Reaction Network Data set	2011	Network of metabolic pathways. A reaction network and a relation network.	65,554
OASIS-2	2010	Longitudinal MRI data in nondemented and demented older adults	150
Diabetic Retinopathy Messidor Data set	2008	Methods to evaluate segmentation and indexing techniques in the field of retinal ophthalmology (MESSIDOR).	1200
Diabetic Retinopathy Messidor Data set	2008	Methods to evaluate segmentation and indexing techniques in the field of retinal ophthalmology (MESSIDOR)	1200
P300 Interface Data set	2008	Data from nine subjects collected using P300-based brain-computer interface for disabled subjects.	1224
OASIS-1	2007	Cross-sectional MRI data in young, middle aged, nondemented and demented older adults	416
OASIS-3	2005	Longitudinal neuroimaging, clinical, and cognitive data set for normal aging and Alzheimer’s disease	1098
EEG Database	1999	Study to examine EEG correlates of genetic predisposition to alcoholism.	122
Arrhythmia Data set	1998	Data for a group of patients, some of whom have cardiac arrhythmia.	452
Breast Cancer Wisconsin Data set	1995	Data set of features of breast masses. Diagnosis by physician is given.	569
Lung Cancer Data set	1992	Lung cancer data set without attribute definitions.	32
Liver Disorders Data set	1990	Data for people with liver disorders.	345
Heart Disease Data Set	1988	Attributes of patients with and without heart disease.	303
Thyroid Disease Data set	1987	10 databases of thyroid disease patient data.	7200

Many companies use TensorFlow: Google, Coca-Cola, Airbnb, Intel, Twitter, LinkedIn, Airbus, eBay, Lenovo, PayPal, Dropbox, Uber, AMD, and DeepMind, just to name a few. But is TensorFlow and Keras also used in medical science? Of course. More and more every day. Not only that, the use of TensorFlow in medical research has led to further improvements to this machine/deep learning framework. Below are a few fresh examples.

Zhang and Kagen (1) constructed a single-layer artificial neural network in TensorFlow to classify healthy individuals vs Parkinson’s disease patients based on 1513 Digital Imaging and Communications in Medicine (DICOM) standard images. The authors expanded and refined the TensorFlow API to make it compatible with the DICOM standard, which allowed the further use of TensorFlow in the work with image sets of that standard. Grover et al (2) predicted the severity of Parkinson’s disease by a deep neural network (DNN) constructed using the TensorFlow/Keras framework. Based on 5875 instances, they showed that DNN produced much better results in severity assessment than other techniques. In addition, a classification based on the Unified Parkinson’s Disease Rating Scale (UPDRS) motor score was better than the classification based on the UPDRS total score and, therefore, it could be used as a better metric for severity prediction.

There is an extremely strong motivation for finding better and more reliable diagnostic methods for breast cancer. Cogan et al (3) created a complete screening solution with three primary parts: a service to upload images and review results, machine learning algorithm to accept or reject images as valid mammograms, and artificial neural network to locate prospective malignancies. They used the Digital Database for Screening Mammography (DDSM) and the INbreast database for training and validation using the TensorFlow Object Detection API for implementing the network. Similar work aimed at risk stratification in breast cancer was done by Ha et al in 2019 (4), who used 1474 mammograms. A model based on the convolutionary neural network (CNN) in the TensorFlow framework showed significantly greater predictive potential compared with traditional prediction according to breast density. CNNs represent state-of-the-art for complex classification problems.

In 2017, Lopez-Rincon et al (5) used CNN in the TensorFlow framework for cancer miRNA biomarkers classification. Although their results were only preliminary, based on 1046 biomarkers for 8129 patients affected by 29 different types of cancers, they showed which tumor classes were more difficult to detect, and indicated the cancer types for which miRNA might be a valuable biomarker. Also, using CNNs, Riordon et al (6) showed deep learning to be an effective means for classifying sperm without the pre-extraction of shape descriptors, relying uniquely on image inputs.

TensorFlow was also used in the work by Dimauro et al 2019 (7) for CNN creation and classification of rhino-cytograms. Although they had only fourteen slides available, they created a data set of 12 298 (587 fields were taken from all available slides). A total of 3423 cells were manually selected and labeled after an image cleaning process in order to generate the basic data set. Based on this data set of relatively poor origin, the authors demonstrated the appropriateness of CNN use in the analysis of rhino-cytograms.

A DNN created using the TensorFlow framework was used to predict asthma severity as well as the likelihood of an asthmatic attack (8). The results of this study are based on an enhanced artificial neural network model with multi-layer perception that can predict a possible asthmatic attack with a remarkable degree of accuracy. Tomita et al (9) demonstrated the benefits of DNN in the TensorFlow framework, with an input layer composed of 22 nodes, for diagnosing adult asthma in comparison with machine-learning based methods.

Attia et al (10) developed an AI-enabled electrocardiograph to identify the signature of atrial fibrillation during normal sinus rhythm using a CNN built within the TensorFlow/Keras framework. Based on the use of the data set obtained from 649 931 ECGs from 180 922 patients, they found that such advanced “AI-enabled electrocardiograph” allows the identification of patients with atrial fibrillation at the point of care.

In 2019 (11), Ting et al demonstrated how deep learning, ie CNN, can be applied in ophthalmology, while Erdenebayer et al (12) employed as many as six different deep learning models designed using the Keras library with the TensorFlow background to automatically detect sleep apnea events from electrocardiograms.

As predicting the binding affinity of major histocompatibility complex I (MHC I) proteins is important for vaccine design, O'Donnell et al (13) developed an open source package for MHC I binding prediction, named MHCflurry, based on the Keras neural network library implemented with Python program language.

Augusta et al (14) showed how to use deep learning for the classification of spatial epidemics. For this purpose, the authors used the Keras library within the statistical environment R rather than Python.

There are many more examples of the increasing use of machine/deep learning methods in medical laboratory and clinical research, primarily due to the efforts of software developers to improve APIs in high-level computer languages. Currently, perhaps the best example is the Keras high-level framework with the low-level TensorFlow background.

The mentioned examples of the use of the TensorFlow and Keras machine/deep learning framework show that this concept is applicable to all sizes of data sets – from a relatively small number of patients to hundreds or hundreds of thousands of patients. At first glance, it may seem that machine/deep learning methods are most widely used in neurology, ie, in those areas of medicine where image analysis is important. However, the examples from oncology, pulmonology, ophthalmology, cardiology, clinical molecular biology, immunology, and epidemiology show that AI data processing techniques are applicable in all branches of medicine that deal with a greater amount of data. This is one of the reasons why the organization of databases and their storage is crucial at all levels of clinical structures, from cytology and biochemistry laboratories to clinic and hospital wards.

Finally, we should cite Fourcade and Khonsari (15), who concluded that medical doctors now have a historic opportunity to participate in a scientific revolution by understanding deep learning, by participating in the design and assessment of new devices and tools, and by contributing to the creation of a framework for regulating this new form of medical practice.
